# Cytokine Profiles and Cell Proliferation Responses to Truncated ORF2 Protein in Iranian Patients Recovered from Hepatitis E Infection

**DOI:** 10.1155/2015/523560

**Published:** 2015-09-15

**Authors:** Reza Taherkhani, Fatemeh Farshadpour, Manoochehr Makvandi, Hamid Rajabi Memari, Ali Reza Samarbafzadeh, Nasrin Sharifi, Behrouz Naeimi, Saeed Tajbakhsh, Samad Akbarzadeh

**Affiliations:** ^1^Department of Microbiology and Parasitology, School of Medicine, Bushehr University of Medical Sciences, Bushehr 7514633341, Iran; ^2^Persian Gulf Tropical Medicine Research Centre, Bushehr University of Medical Sciences, Bushehr 7514633341, Iran; ^3^Health Research Institute, Infectious and Tropical Disease Research Centre, Ahvaz Jundishapur University of Medical Sciences, Ahvaz 6135715794, Iran; ^4^Department of Agronomy and Plant Breeding, Faculty of Agriculture, Ahvaz Shahid Chamran University, Ahvaz 6135715794, Iran; ^5^Department of Nutrition, School of Medicine, Kashan University of Medical Sciences, Kashan 8715988141, Iran

## Abstract

*Background.* The aim of this study was to evaluate* hepatitis E virus* (HEV) specific cellular immune responses to truncated ORF2 protein in Iranian patients recovered from HEV infection. Information about HEV-specific immune responses could be useful in finding an effective way for development of HEV vaccine.* Methods. *A truncated form of HEV ORF2 protein containing amino acids 112-608 was used to stimulate peripheral blood mononuclear cells (PBMCs) separated from HEV-recovered and control groups. Finally, the levels of four cytokines, IFN-*γ* ELISPOT, and cell proliferative responses following stimulation with the truncated ORF2 protein were assessed in the both groups.* Results.* The truncated ORF2 protein was able to induce IFN-*γ* ELISPOT and cell proliferation responses and to produce significant amounts of IFN-*γ* and IL-12 cytokines, but low amounts of IL-10 and IL-4 cytokines* in vitro*. These responses were significantly higher in the recovered group compared to the control group. These results indicate the antigenic nature of the truncated ORF2 protein and production of T helper type 1 cytokines.* Conclusion. *The truncated ORF2 protein can effectively induce significant cellular immune responsesand can be introduced as a potential vaccine candidate. However, further studies are required to evaluate this protein* in vivo*.

## 1. Introduction

*Hepatitis E virus* (HEV) is a nonenveloped virus with a nonsegmented positive-sense RNA genome containing three open reading frames (ORFs) [1, 2]. HEV belongs to the family Hepeviridae and the genus* Hepevirus* [3]. The genus* Hepevirus* has two species including mammalian HEV and avian HEV. The mammalian HEV, which infects humans and several mammalians, is classified into four major genotypes, namely, genotypes 1, 2, 3, and 4 [4]. The HEV genotypes share highly conserved immunodominant domains that are serologically cross-reactive [5].

HEV causes large outbreaks of acute hepatitis in developing countries where sanitary conditions are inadequate and the infection is enterically transmitted by the fecal-oral route especially contaminated water [6, 7]. The zoonotic spread of the disease is also possible especially in developed countries where the sporadic cases of the infection have been reported [2, 4]. Although the infection is usually asymptomatic or acute self-limiting in general population, but it can progress to lethal fulminant hepatitis in pregnant women and chronic hepatitis in immunosuppressed and organ transplant patients [5, 8]. The general mortality rate of HEV infection is between 1% and 15%, and this level may reach up to 30% due to fulminate hepatic failure during pregnancy, which may lead to high rate of spontaneous abortion and premature birth [5, 9]. Consequently, HEV has become a serious public-health concern especially in endemic areas, and control and prevention of this infection are necessary [2]. So far, no effective treatment or commercial vaccines for HEV infection are available, and the only way to reduce incidence of the infection is prevention [2, 10].

Currently, the focus for development of HEV vaccine is on ORF2 protein. ORF2 encodes capsid protein that comprises 660 amino acids. HEV capsid protein has several immune-dominant conserved epitopes that can induce long-lived immunity; therefore, it has been studied for vaccine development [11]. However, the full-length capsid protein is not suitable for vaccine production. Since it is hydrophobic and insoluble and its immunoreactive epitopes are masked, much attention has been drawn to the truncated or short forms of ORF2 protein as protective vaccines [12, 13].

The truncated ORF2 protein, containing amino acids 112 to 660 of the ORF2 protein, self assembles into virus-like particles and can induce strong immunity [14]. Moreover, 52 amino acids from the C terminus and 111 amino acids from the N terminus of the ORF2 protein do not have any antigenic domain, and most antigenic domains have located within 268 amino acids of C terminal of the ORF2 protein [5]. Also, the truncated ORF2 protein containing amino acids 459 to 607 is needed to produce anti-HEV antibodies; therefore, it seems that antigenic epitopes are conformational [5]. Anti-HEV antibodies from all genotypes cross-react with the ORF2 protein of genotype 1 [5]; therefore, production of a broadly protective vaccine seems to be possible by expression the truncated ORF2 protein of genotype 1 [13].

In the present study, the cellular immune responses to the truncated ORF2 protein were evaluated in the peripheral blood mononuclear cells (PBMCs) of individuals recovered from hepatitis E infection.

## 2. Materials and Methods

### 2.1. Ethics Statement

This study (with research project number D/574) was approved by the Jundishapur University Ethical Committee, and informed consent was taken from all the participants.

### 2.2. Study Group

Forty anti-HEV IgG positive subjects (24, 60% males and 16, 40% females; mean age ± SD, 35.12 ± 9.66 years) and forty-eight anti-HEV IgG negative subjects (28, 58.3% males and 20, 41.7% females; mean age ± SD, 33.06 ± 10.70 years) were randomly selected as HEV-recovered and control groups to study the immunogenicity of the truncated ORF2 protein. The presence of anti-HEV IgG antibody was detected by the commercial ELISA kit (HEV IgG ELISA kit, DIA.PRO, Milan, Italy). All the participants were negative for anti-HEV IgM antibody, anti-HAV IgM antibody, anti-HCV antibodies, and hepatitis B surface antigen (HEV IgM ELISA kit, HAV IgM ELISA kit, HCV Ab ELISA kit, HBs Ag ELISA kit, DIA.PRO, Milan, Italy) and had normal ALT level.

### 2.3. Preparation of Peripheral Blood Mononuclear Cells

Ficoll-Hypaque (Lymphoflot, Biotest Diagnostics, Germany) and density gradient centrifugation method were used to separate peripheral blood mononuclear cells (PBMCs) from 10 mL heparinized venous blood sample of the each individual. Then the cells were washed twice by RPMI 1640 medium and resuspended in RPMI 1640 plus 10% FCS (Invitrogen, Carlsbad, CA, USA). Trypan blue staining was used to evaluate the cells viability.

### 2.4. Preparation of Truncated ORF2 Protein

A truncated form of HEV ORF2 protein containing amino acids 112–608 was produced as described previously [15]. Briefly, a truncated ORF2 gene encoding amino acids 112–660 of Sar-55 strain from HEV genotype 1 was optimized for expression in* E. coli* and synthesized and cloned into pBluescript II SK(+) vector by Biomatik Company (Biomatik Corporation, Cambridge, Canada). The truncated gene was subcloned into expression vector pET-30a(+) and the recombinant plasmid pET30a-ORF2_(aa 112–660)_ was constructed. Then, a 193-nucleotide fragment was removed from the nucleotide sequence of the truncated ORF2 gene by digestion with NheI restriction enzyme and another truncated form of ORF2 gene encoding amino acids 112–608 was constructed. The recombinant plasmid pET-30a-ORF2_(aa 112–608)_ was transformed into* E. coli* BL21 (DE3) using electroporation. Expression of the truncated ORF2 protein containing amino acids 112–608 was induced by adding 1 mM IPTG to the bacterial culture followed by 4 hr incubation at 37°C. The expressed truncated ORF2 protein was purified by Ni2+-chelate-affinity chromatography (Qiagen, Hilden, Germany). Refolding of the purified protein was done by dialysis in PBS + 10% glycerol at 4°C for 4 hr. Amicon Ultra-4 Centrifugal Filter Unit (EMD Millipore, Billerica, MA, USA) was used to improve the concentration and purity of the target protein. To remove endotoxin contamination that may be obtained during protein purification, endotoxin was extracted from the protein by Toxin Eraser Endotoxin Removal kit (GenScript, Piscataway, NJ, USA).

### 2.5. Cell Proliferation Assay

The cell proliferation was carried out by the colorimetric assay using MTT (3-(4,5-dimethylthiazol-2-yl)-2,5-diphenyl tetrazolium bromide) as described previously [16]. For each sample, approximately 1 × 10^5^ PBMCs/well in RPMI 1640 plus 10% FCS were added to 3 wells of 96-well plates in final volume of 180 *μ*L/well. The first well was stimulated with 10 *μ*g/mL of truncated ORF2 protein in final volume 20 *μ*L. 20 *μ*L/well of phytohemagglutinin (PHA) (5 *μ*g/mL) (Sigma–Aldrich, St. Louis, MO, USA) was used as positive control in the second well and the third well remained unstimulated as negative control. Blanking was done by putting RPMI 1640 (20 *μ*L) alone in the well. The optimal concentrations of truncated ORF2 protein and PHA and the optimum stimulation duration were determined based on preliminary experiments. The plate was incubated in the presence of 5% CO_2_ at 37°C for 4 days, and then 10 *μ*L of MTT solution (5 mg/mL) was added to each well. After incubation at 37°C for 4 hr and centrifugation in 1000 ×g for 10 min, the supernatant was discarded and 100 *μ*L of DMSO was added to each well and mixed by pipetting. The absorbance of the wells was measured using an ELISA reader at 570 nm within an hour. The results were expressed as proliferation index (PI). PI was defined as OD570 stimulated sample/OD570 unstimulated sample.

### 2.6. IFN*γ* ELISPOT Assay

The IFN*γ* ELISpot assay was performed by Human IFN-*γ* ELISPOT Ready-SET-Go kit (eBioscience, San Diego, CA, USA) to determine HEV-specific IFN-*γ*-producing cells. According to manufacturer's structure, a PVDF bottomed ELISPOT plate (Millipore, Bedford, MA, USA) was coated with Capture Antibody solution (100 *μ*L/well) overnight at 4°C. For each sample, approximately 1 × 10^5^ PBMCs/well in RPMI 1640 plus 10% FCS were added to 3 wells of the 96-well ELISPOT plate in final volume of 150 *μ*L/well. The first well was stimulated with 10 *μ*g/mL truncated ORF2 protein. Five *μ*g/mL of PHA was used as positive control in the second well, and the third well remained unstimulated as negative control. The optimal count of PBMC in each well, the optimal concentrations of truncated ORF2 protein and PHA, and the optimum duration of stimulation were determined based on preliminary experiments. The plate was incubated at 37°C for 24 hr. Then, the cells and medium were removed and the wells were washed 3 times with ELISPOT Wash Buffer. The plate was incubated with Biotinylated Detection Antibody (100 *μ*L/each well) at 4°C overnight and washed 4 times with ELISPOT Wash Buffer. In the next step, the plate was incubated with 100 *μ*L/well of Avidin-Horseradish Peroxidase reagent at room temperature for 45 min and washed with ELISPOT Wash Buffer and 1x PBS. Finally, the dark purple spots were developed by adding 100 *μ*L/well AEC (3-amino-9-ethylcarbazole), followed by incubation at room temperature. The plate was washed 3 times with 200 *μ*L/well distilled water and dried at room temperature, and then a dissection stereoscope was used to count the number of spots. The results were shown as spot forming cells (SFCs) per 10^5^ cells, while each spot was considered as one HEV-specific IFN-*γ* secreting cell.

### 2.7. Cytokine Assay

PBMCs of each sample were stimulated with the truncated ORF2 protein (10 *μ*g/mL) at 37°C. In addition, five *μ*g/mL PHA was used as positive control. The optimal concentrations of truncated ORF2 protein and PHA and the optimum stimulation duration were determined based on preliminary experiments. Culture supernatants were harvested after 1-2 days and used to measure amounts of four cytokines (IL-4, IL-12 p70, IFN-*γ*, and IL-10). Cytokine assay was performed by commercially available ELISA kits (eBioscience, San Diego, CA, USA) according to the manufacturer's instructions. The results were shown as picograms per milliliter (pg/mL). The limits of sensitivity of the assays were 1.3 pg/mL for IL-4, 1.0 pg/mL for IL-10, 2.1 pg/mL for IL-12 p70, and 0.99 pg/mL for IFN-*γ*.

### 2.8. Statistical Analysis

SPSS 17 Package program (SPSS Inc., Chicago, USA) was used for statistical analysis, and *p* values below 0.05 indicated statistical significance. The data were analyzed and compared by the Mann-Whitney *U* test or independent *t*-test. All data were expressed as mean ± SD.

## 3. Results

### 3.1. Cell Proliferative Response to Truncated ORF2 Protein

**Figure 1:**
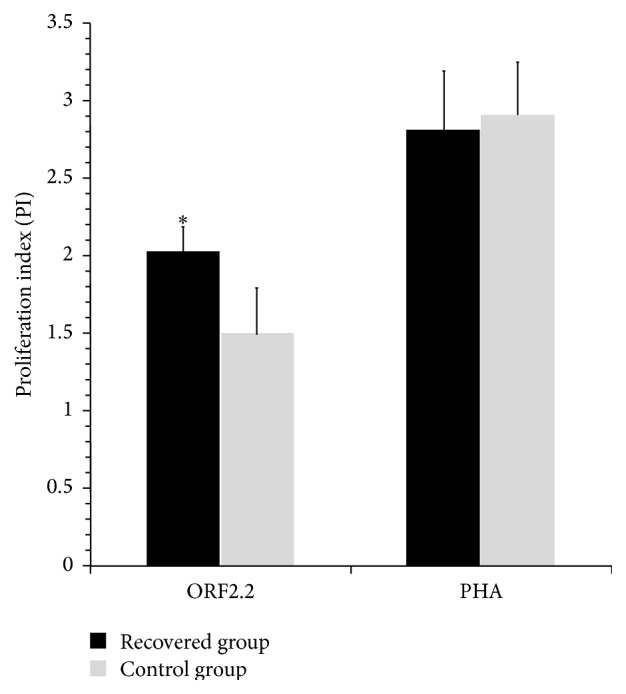
Cell proliferative responses in HEV-recovered individuals and control group. Proliferative responses to the truncated ORF2 protein are significantly higher in HEV-recovered group compared to the control group, while both groups showed good nonspecific stimulation with PHA. The results are shown as proliferation index (mean ± SD PI).

Proliferation of PBMCs in response to the truncated ORF2 protein was measured in the control and HEV-recovered groups. The cell proliferative responses to truncated ORF2 protein in the control and HEV-recovered groups were 1.50 ± 0.17 and 2.01 ± 0.28, respectively. The corresponding figures following stimulation with PHA were 2.91 ± 0.37 and 2.81 ± 0.33 in the control and recovered groups, respectively (*p* = NS). Overall, the recovered group showed significantly higher cell proliferation compared to the control group (*p* < 0.001) (Table 1, Figure 1).

### 3.2. IFN-*γ* ELISPOT Responses to the Truncated OR2 Protein

**Figure 2:**
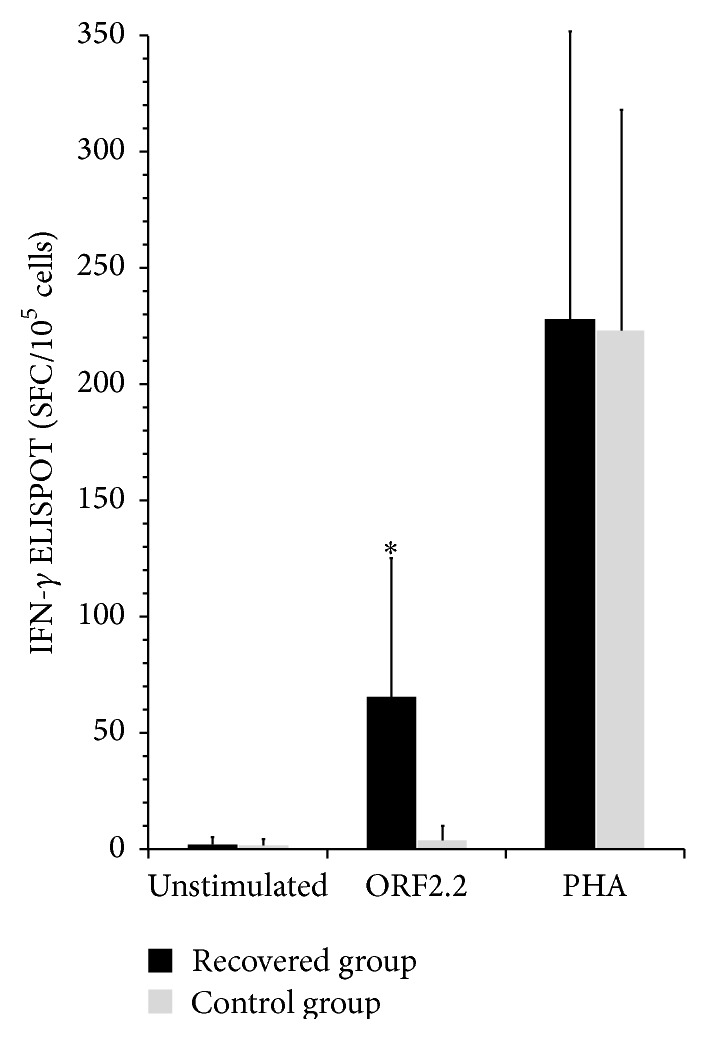
IFN-*γ* ELISPOT responses in HEV-recovered and control groups. IFN-*γ* ELISPOT responses to the truncated ORF2 protein are significantly higher in the recovered group compared to the controls, while both groups showed good nonspecific stimulation with PHA. The results are shown as spot forming cells per 10^5^ cells (SFC/10^5^ cells).

The frequencies of IFN-*γ*-secreting cells in PBMCs of HEV-recovered and control groups were assessed in response to truncated ORF2 protein by ELISPOT assay. IFN-*γ* ELISPOT responses following stimulation with the truncated ORF2 protein in the control and HEV-recovered groups were 4.39 ± 5.51 and 65.92 ± 58.99, respectively. ELISPOT responses to PHA in the control and recovered groups were 223.41 ± 94.58 and 228.25 ± 123.41, respectively (*p* = NS). Overall, IFN-*γ* ELISPOT responses were significantly higher in the recovered group compared to the control group (*p* < 0.001) (Table 1, Figure 2).

### 3.3. Cytokine Production in Responses to Truncated ORF2 Protein

**Figure 3:**
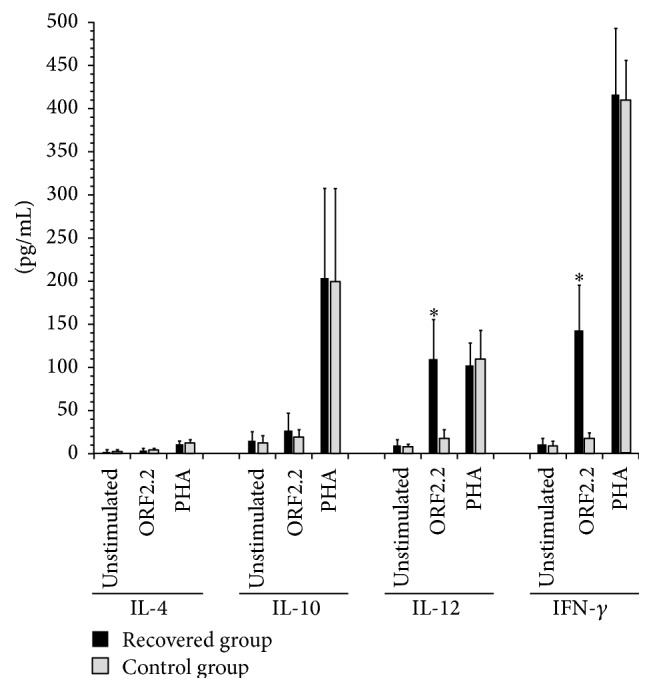
The levels of IL-4, IL-10, IL-12 p70, and IFN-*γ* cytokines in HEV-recovered and control groups following stimulation with the truncated ORF2 protein. Significant amounts of IFN-*γ* and IL-12 cytokines but low amounts of IL-10 and IL-4 cytokines were produced. IL-12 p70 and IFN-*γ* cytokines production following stimulation with the truncated ORF2 protein was significantly higher in the recovered group compared to the control group, while there were no significant differences in the levels of IL-10 and IL-4 between the two groups. Both groups showed almost similar expression of all cytokines following stimulation with PHA. Results are shown as mean ± SD pg/mL.

Significant amounts of IFN-*γ* and IL-12 cytokines but low amounts of IL-10 and IL-4 cytokines were produced following stimulation with the truncated ORF2 protein. Comparison between the two groups revealed no significant differences in the levels of IL-10 (*p* = 0.100) and IL-4 (*p* = 0.067), while IFN-*γ* and IL-12 p70 were significantly higher in the recovered group compared to the control group (*p* < 0.001). Stimulation with PHA resulted in almost similar expression of all cytokines in the control and recovered groups, including 11.96 ± 4.55 and 11.29 ± 3.9 for IL-4 (*p* = NS), 199.45 ± 108.43 and 204.97 ± 103.61 for IL-10 (*p* = NS), 109.91 ± 32.86 and 103.22 ± 25.9 for IL-12 (*p* = NS), and 410.37 ± 46.96 and 418.37 ± 75.65 for IFN-*γ* (*p* = NS), respectively (Table 1, Figure 3).

## 4. Discussion

HEV causes self-limited acute hepatic infection worldwide and especially in developing countries where the disease is endemic and 30% to 80% of the adult population has anti-HEV antibody [17]. The infection is more severe in pregnant women and patients with underlying liver problems and may progress to lethal fulminant hepatitis [5, 8]. Information about HEV specific cellular immune responses could be useful in finding an effective way for development of HEV vaccine, in particular as any protective vaccine for HEV infection is not still licensed [2, 9]. However, some studies have evaluated HEV-specific cellular immune responses among different groups [6, 18–20], but current information on cellular immune responses against HEV is not sufficient to prevent HEV infection. Therefore, this study aimed to evaluate HEV-specific cellular immune responses to the truncated ORF2 protein in the recovered individuals from HEV infection.

In the present study, a significant increase in the cell proliferation and IFN-*γ* ELISPOT responses following stimulation with the truncated ORF2 protein were found in the HEV-recovered subjects, while both groups showed good nonspecific stimulation with PHA. Therefore, the increase in responses induced by truncated ORF2 protein was specific in the recovered group. These results indicate the antigenic nature of the truncated ORF2 protein. Suneetha et al. reported a similar increase in the HEV-specific T-cell responses by PBMCs obtained from recovered individuals from HEV infection. The stimulating antigen in their study was overlapping peptide pools and not the whole HEV ORF2 protein [20]. Regarding the full length ORF2 protein, Husain et al. presented similar results in acute hepatitis E patients compared to the controls [21]. It has been shown that ORF2 protein has several HTL (helper T-lymphocyte) dominant immunogenic regions and therefore it is a good antigen to elicit high and long-lasting specific anti-HEV immune responses [22]. The HEV-specific T-helper cell responses, especially against capsid protein, play a critical role in clearing and controlling viral infections, either directly through cytokines production or indirectly by activating cytotoxic T cells [1].

Production of T-helper type 1 (Th1) cytokines such as IFN-*γ*, TNF-*α*/*β*, IL-2, IL-3, and IL-12 have been implicated in viral clearance or resistance to infection, while production of Th2 cytokines such as IL-4, IL-5, IL-13, IL-6, and IL-10 cytokines is associated with progression of the infection or viral persistence [23–28]. In the present study, the truncated ORF2 protein could significantly stimulate PBMCs to produce IFN-*γ* and IL-12 but little or no IL-4. These results demonstrate that the Th1 responses are induced against the truncated ORF2 protein. The results of this study being consistent with those of others suggest that viral proteins that can shift immune responses to Th1, rather than Th2, may have a better chance of success as a vaccine candidate against HEV infection [29]. All participants in control and recovered groups showed almost similar expression of all cytokines following stimulation with PHA. Therefore, the increase in production of IFN-*γ* and IL-12 cytokines following stimulation with truncated ORF2 protein was specific in the recovered group.

The results of the previous studies have shown that the production of Th1 cytokines such as IL-12 and IFN-*γ* during HEV infection results in self-limiting hepatitis E, suggesting the role of these cytokines in the recovery and clearance of hepatitis E, while the Th2 cytokines increase in fatal fulminant hepatitis E, especially during pregnancy [18, 27, 30, 31]. In Saravanabalaji et al. study, levels of IFN-*γ* and IL-12 cytokines following stimulation of PBMCs by recombinant ORF2 protein were higher in recovered patients than those with fatal fulminant hepatitis E, while IL-10 was higher in fatal patients [30]. In Pal et al. study, high levels of IL-4 and IL-10 were observed in pregnant women with acute hepatitis E following stimulation of PBMCs with different peptides of HEV ORFs, while the level of IFN-*γ* was low in this group [27]. In Srivastava et al. study, the IFN-*γ* production following stimulation with ORF2 protein was higher in patients with acute HEV infection compared to control [6].

In this study, antigenic properties of the expressed ORF2 protein in* E. coli* BL21 cell were evaluated. Among the various microorganisms and systems available for protein production,* E. coli* BL21 containing pET-derived plasmids has become the most commonly used expression system because of its high expression capabilities, protein production with high purity, low cost, simple and fast cultivation, its well-known genetics, and therefore ease of genetic manipulation [32]. However, there are a variety of factors that affect the expression level as well as the solubility and especially biological function of overexpressed proteins. One of the ways to improve the expression of foreign protein in* E. coli* is codon optimization, which results in selection of host favorite codons and elimination of high GC contents [33, 34]. By evaluating the antigenic properties and immunogenicity of the truncated ORF2 protein, it was indicated that this protein has biological function and can stimulate specific anti-HEV immune responses.

In conclusion, the truncated ORF2 protein is able to induce significant cellular immune responses and production of Th1 cytokines* in vitro* and therefore can be introduced as a potential vaccine candidate for HEV infection. However further studies are required to evaluate this protein* in vivo*.

## Figures and Tables

**Table 1:** Results of cytokine, lymphocyte proliferation, and ELISPOT assays in response to truncated ORF2 protein in HEV recovered individuals and control group.

Parameter	Control group (*n* = 48)	Recovered group (*n* = 40)	*p* value
IFN-*γ*	17.12 ± 6.93	143.40 ± 52.33	<0.001
IL-12	18.33 ± 9.04	112.75 ± 43.26	<0.001
IL-10	18.72 ± 8.55	27.80 ± 19.55	0.100 (NS)
IL-4	4.30 ± 1.53	4.90 ± 1.49	0.067 (NS)
ELISPOT	4.39 ± 5.51	65.92 ± 58.99	<0.001
Cell proliferation	1.50 ± 0.17	2.01 ± 0.28	<0.001

Cytokine values are represented as picograms per milliliter (pg/mL), the results of ELISPOT assay are expressed as spot forming cells per 10^5^ cells (SFC/10^5^ cells), and the results of cell proliferation assay are shown as proliferation index (PI).

All data are shown as mean ± SD.

NS = nonsignificant.
